# Supplementary figures and images for: Sulfite preservatives effects on the mouth microbiome: Changes in viability, diversity and composition of microbiota
Source: PLoS One. 2022 Apr 7;17(4):e0265249. doi: 10.1371/journal.pone.0265249 (PMC8989357; doi:10.1371/journal.pone.0265249)

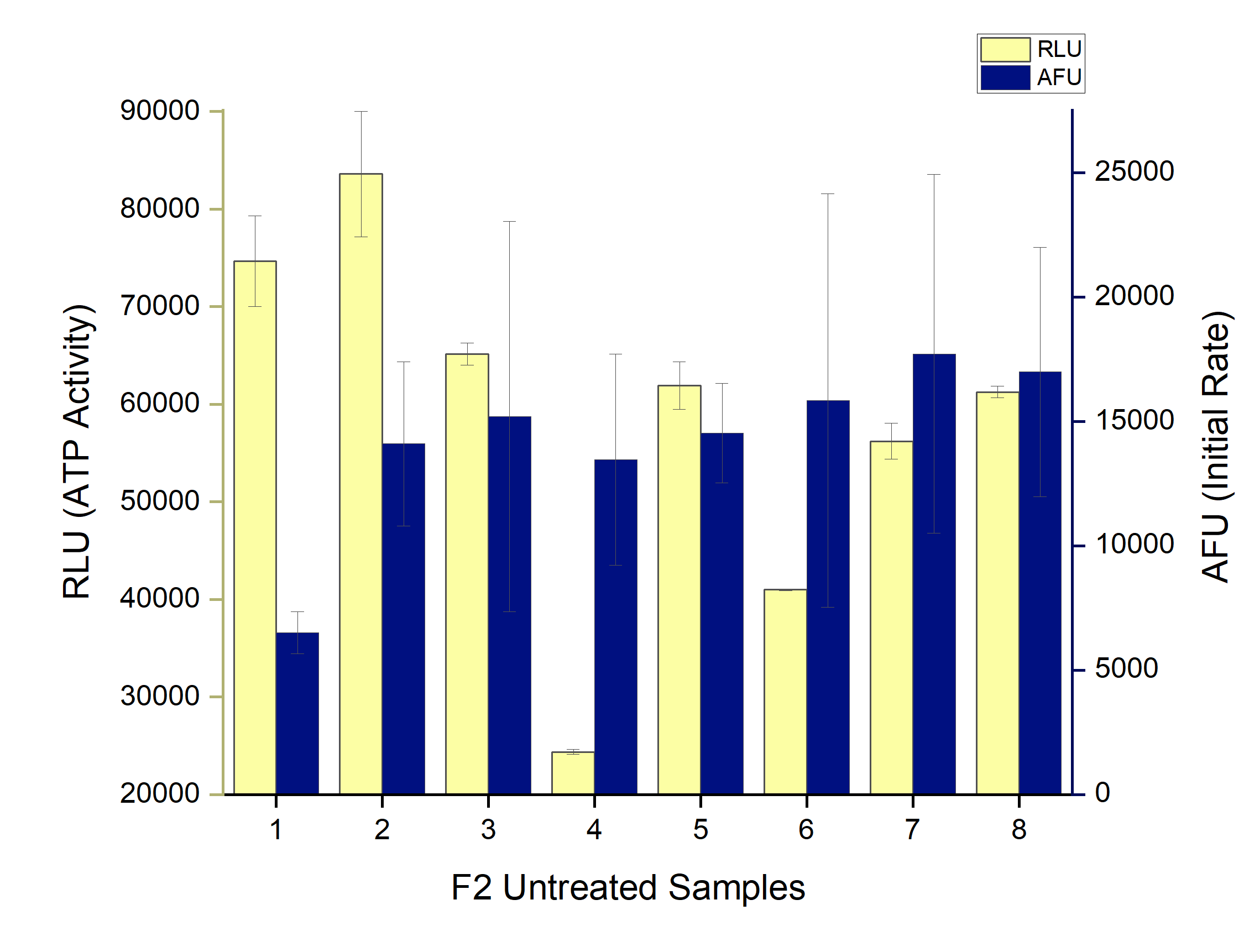

Supplement: S1 Fig — ATP activity in F2 control samples. (TIF) [file pone.0265249.s001.tif]
